# Parent-child nativity, race, ethnicity, and mental health conditions among U.S. children

**DOI:** 10.1192/j.eurpsy.2023.226

**Published:** 2023-07-19

**Authors:** K. Zarei, L. Kahle, D. Buckman, K. Choi, F. Williams

**Affiliations:** 1 National Institute on Minority Health and Health Disparities, Bethesda; 2 Information Management Services, Inc., Calverton, United States

## Abstract

**Introduction:**

Over a quarter of U.S. children have at least one immigrant parent. Mental health disparities in children need to be assessed to better identify disproportionate burdens and promote health equity.

**Objectives:**

To assess the associations between race, ethnicity, and parent-child nativity, and mental health conditions in the U.S.

**Methods:**

Data were from the 2016-2019 National Survey of Children’s Health (n=114,476 children aged 3-17 years), a nationwide, cross-sectional survey. Outcome variables included three mental health conditions (depression, anxiety, and behavior or conduct problems) reported by the parent/guardian. Additional measures included questions about healthcare access and use, demographics, and nine household challenge adverse childhood experiences (ACEs) used to quantify a total ACE score (0-9). Information on nativity was used to define immigrant generation (1^st^, 2^nd^, and 3^rd^+). Weighted logistic regression was used to assess the associations between race/ethnicity (Asian, Black, Hispanic, White, and Other), household generation, and outcome variables, among children who reported access to or utilized health services, adjusting for demographics. Multiple imputation was used to handle missing data.

**Results:**

Asian, Black, Hispanic, and White 3^rd^+ generation children had increased odds of depression compared to their 1^st^ generation counterparts, same as among White, 2^nd^ generation children. Race/ethnicity was not associated with depression among 1^st^ and 3^rd^+ generation children, but Asian, Black, and Hispanic children had lower odds of depression compared to White children among 2^nd^ generation children. Asian, Black, Hispanic, and Other-race 3^rd^+ generation children had increased odds of anxiety compared to their 1^st^ generation counterparts, with similar findings also observed for Black and Other-race 2^nd^ generation children. Being racial/ethnic minorities was generally associated with decreased odds of anxiety among 1^st^ and 2^nd^ generation children compared to White children from the respective generations. Asian, Black, Hispanic, and Other-race 3^rd^+ generation children had increased odds of behavior/conduct problems compared to their 1^st^ generation counterparts. The observed associations remained significant after adjusting for the modified ACE score.

**Conclusions:**

We found significant differences in several mental health conditions in children by parent-child nativity, race, and ethnicity that could not be explained by demographics, childhood adversity, and healthcare access and use. Lower odds of mental health conditions among minority children could represent differences due to factors such as differential reporting, and higher odds of mental health conditions, including in third- and higher generation children, need further investigation to develop approaches to promote mental health equity.

**Disclosure of Interest:**

None Declared

